# The mediating role of unhealthy behavior in the relationship between shift work and perceived health

**DOI:** 10.1186/s12889-021-11350-2

**Published:** 2021-07-02

**Authors:** Karin I. Proper, Eva Jaarsma, Suzan J. W. Robroek, Jolinda L. D. Schram, Hendriek Boshuizen, H. Susan J. Picavet, W. M. Monique Verschuren, Sandra H. van Oostrom

**Affiliations:** 1 Centre for Nutrition, Prevention and Health Services, National Institute for Public Health and the Environment, P.O. Box 1, 3720 BA Bilthoven, the Netherlands; 2 Department of Public and Occupational Health, Amsterdam Public Health Research Institute, Amsterdam UMC, Amsterdam, The Netherlands; 3 Department of Public Health, Erasmus Medical Center, Rotterdam, the Netherlands; 4 Division of Human Nutrition, Wageningen University, Wageningen, the Netherlands; 5 Julius Center for Health Sciences and Primary Care, University Medical Center Utrecht, Utrecht, the Netherlands

## Abstract

**Background:**

Little is known about the relationship between shift work and perceived health, including potential underlying mechanisms such as unhealthy behaviors. The aim of this study was to investigate whether unhealthy behaviors mediate the relationship between shift work and perceived mental and physical health, taking into account potential differences by level of education.

**Methods:**

Data from 1633 workers participating in the Doetinchem Cohort Study during 1995–2016 were used. Being engaged in shift work was determined at 1 year preceding the assessment of health behaviors. Mental and physical health were assessed after 5 years of follow-up by the 5-item Mental Health Inventory and the physical functioning scale of the 36-item Short Form Health Survey. Smoking, physical inactivity, alcohol consumption, and overweight were considered as potential mediators and education was treated as moderator. Moderated mediation analyses using generalized estimated equations were performed.

**Results:**

Shift work was not statistically significantly related to either mental or physical health. Despite this, statistically significant mediation effects of smoking (Beta − 0.09; 95% Confidence Interval − 0.20 - -0.01, respectively B -0.09; 95%CI -0.21 - -0.01) and physical inactivity (B 0.11; 95%CI 0.03–0.23, respectively B 0.08; 95%CI 0.01–0.18) were found in the relationship between shift work and mental or physical health. Direct and indirect effects outweighed each other in the relationship between shift work and mental health, since the direction of these effects was opposite. The relationship between shift work, unhealthy behavior, and health was not different by educational level.

**Conclusion:**

Shift workers did not report lower mental or physical health than non-shift workers. Though mediation effects of unhealthy behavior were observed in the relationship between shift work and perceived health, these small effects had minor public health relevance.

## Background

Today’s 24-h society requires constant production and availability of goods and services [1]. As a result, the number of shift workers has increased in the past decades to make up about 21% of all European workers working in shifts and 19% of all European workers working during the night. Although being employed in shift work is inevitable in some sectors, it can be demanding for health. For example, shift work has been linked to risk factors for health, such as sleep deficits and fatigue [2] and to adverse chronic health effects such as an increased risk of the development of cardiovascular diseases, diabetes type 2, and some types of cancer [3–6]. With shift work becoming more common, this may reflect a public health concern.

Not much is known about the effects of shift work on perceived health, including general, mental, and physical health. There is a minimal amount of literature available on the relationship between shift work and perceived health, and results of those studies were mixed [7–10]. For example, data from the Korea Health Panel of working women, showed that night workers and rotating shift workers had lower health-related quality of life scores compared to those working day shifts [10]. In contrast, another cross-sectional study found a better general health status [8], and some other studies did not find differences in perceived health between shift and non-shift workers [7, 9]. For mental health, the limited studies available were inconsistent in their results as well. Some studies found shift work to be associated with anxiety, depression or low general mental health [6, 11–13], while others observed favorable effects [14] or no associations [9, 15, 16]. Considering the predictive value of perceived health on morbidity and mortality [17], more research into the effect of shift work on perceived health is needed.

Several pathways have been proposed to explain the potential negative health effects of shift work, including unhealthy behaviors [4, 18–21]. Regarding physical activity levels, some studies showed that shift workers are less physically active in leisure time compared to non-shift workers [22, 23], suggesting a potential mediating role of this behavior. However, other studies did not support these findings, and even found higher levels of walking activity in shift workers and similar leisure time physical activity compared to non-shift workers [24, 25]. Also, for other unhealthy behaviors, such as smoking and alcohol consumption, the evidence for a link with shift work has been mounting [6]. For example, a systematic review of six longitudinal studies concluded that smoking was more frequent among shift workers [26].

Knowledge about the mechanistic (mediating) factors in the effects of shift work on perceived health is necessary to provide opportunities for improving health in shift workers and to prevent future adverse health effects. The mediating role of unhealthy behaviors in the relation between shift work and perceived health has not been tested so far. It has been shown that both unhealthy behaviors and unfavorable working conditions, such as shift work, are more common among workers with a lower educational level than among workers with a higher educational level [27]. Also the adverse health effects of unhealthy behaviors and unfavorable working conditions might differ according to level of education. It is therefore important to take into account possible moderation by educational level. The aim of this study was to investigate whether unhealthy behaviors mediate the relationship between shift work and perceived (mental and physical) health, taking into account possible moderation by educational level.

## Methods

Data from the longitudinal Doetinchem Cohort Study (DCS) were used, which is an ongoing population-based study of men and women who were aged 20–59 years at the start of the study. Participants were examined in 1987–1991 (*N* = 7768), 1993–1997 (*N* = 6117), 1998–2002 (*N* = 4918), 2003–2007 (*N* = 4520), 2008–2012 (*N* = 4018) and 2013–2016 (*N* = 2798). Response levels were 75% or higher over the different measurement rounds [28, 29]. Informed consent was obtained from all individual participants.

Data from the period 1994–2016 were used, since the relevant outcome measures were available over this period. We included 2798 participants who participated in round 6 (2013–2016) because this round included questions about lifetime shift work history, which enabled us to retrospectively determine shift work status at previous assessment waves. We excluded participants who never had a paid job (*N* = 1062). Those who did but stopped doing paid work and did not return to paid work before the sixth round were excluded from the analyses from the moment they stopped doing paid work. Of the participants who filled out the questionnaire about shift work, 1736 reported doing paid work in at least one of the rounds 2 to 5. There were 103 respondents who filled out the shift work questionnaire incompletely or provided inconsistent answers, making it impossible to establish the period in which they did shiftwork. These respondents were excluded. Of the 1633 remaining participants, 232 reported doing shift work at least once during the relevant rounds. In total 625 shift work observations were available over the four measurement rounds. Of those, 195 participants were shift workers at the year preceding their baseline assessment.

### Perceived health

Perceived health was operationalized as self-rated mental and physical health. Mental health was assessed by the 5-item Mental Health Inventory (MHI-5), which includes 5 items on depressive and anxiety symptoms, like nervousness and feeling down. Items were scored on a 6-point Likert scale ranging from ‘all of the time’ to ‘none of the time’. The physical functioning scale of the 36-item Short Form Health Survey (SF-36) was used as a proxy for physical health and contains 10 items on physical activities, daily routine activities (ADL’s) and “instrumental” activities (IADL’s), such as walking 500 m or washing yourself. Items in the physical health scale had 3 response options: yes, limited a lot; yes, limited a little; no, not limited at all. Scores on both the mental and physical scale were summed and rescaled to a score from 0 to 100, where higher scores indicate better perceived health [30–32].

### Shift work

A retrospective questionnaire about shift work was included in the sixth round of the DCS, including the most important aspects of shift work [33]. Shift work was defined as working evening shifts (i.e. shifts ending before midnight), night shifts (i.e. shifts starting at or after midnight), and/or rotating shifts (i.e. rotating between day, evening and/or night shifts). Therefore, participants were asked whether they had ever done evening, night or rotating shifts. If so, they were asked to indicate the total number of years they had done shift work, the year they started and the year they stopped doing shift work [34]. To ensure that shift work preceded unhealthy behavior in the analyses, it was determined whether participants did shift work in the year preceding each measurement.

### Body mass index and unhealthy behavior

Body mass index (BMI) was based on objective measurements of body height and weight at each measurement round. BMI was dichotomized into normal weight (< 25 kg/m^2^) and overweight (≥ 25 kg/m^2^). Smoking, alcohol consumption and physical activity were included in the questionnaire at each measurement round of the DCS. Smoking status was based on the question ‘do you smoke cigarettes?’ and dichotomized into current smokers and non-smokers. Alcohol consumption was measured by asking participants to indicate how many glasses of alcoholic beverages they drank per week. This number was converted into an average number of glasses of alcoholic beverages per day. Alcohol consumption was dichotomized as high (> 1 glass per day) or low (≤ 1 glass per day), based on the Dutch dietary guidelines [35]. Physical activity in leisure time was assessed by a questionnaire, developed for the EPIC study [36]. Physical activity was dichotomized based on adherence or not to the Dutch guideline for physical activity, i.e. being physically active with moderate or high intensity for an average of 30 min per day [37]. Instead of the recommended 2.5 h a week spent on at least moderately intense physical activity, we used a cut-off point of 3.5 h a week to define physically active and physically inactive workers [38]. This is justified to account for the facts that (i) we had only data averaged over a week and (ii) that the amount of activity is often overreported [38].

### Educational level

Level of education was assessed as the highest level achieved, as reported in round 2. If missing, data from round 3 were used. Educational level was classified as either low (intermediate secondary education or less) or intermediate/high (intermediate vocational and higher secondary education or higher).

### Statistical analyses

**Fig. 1:**
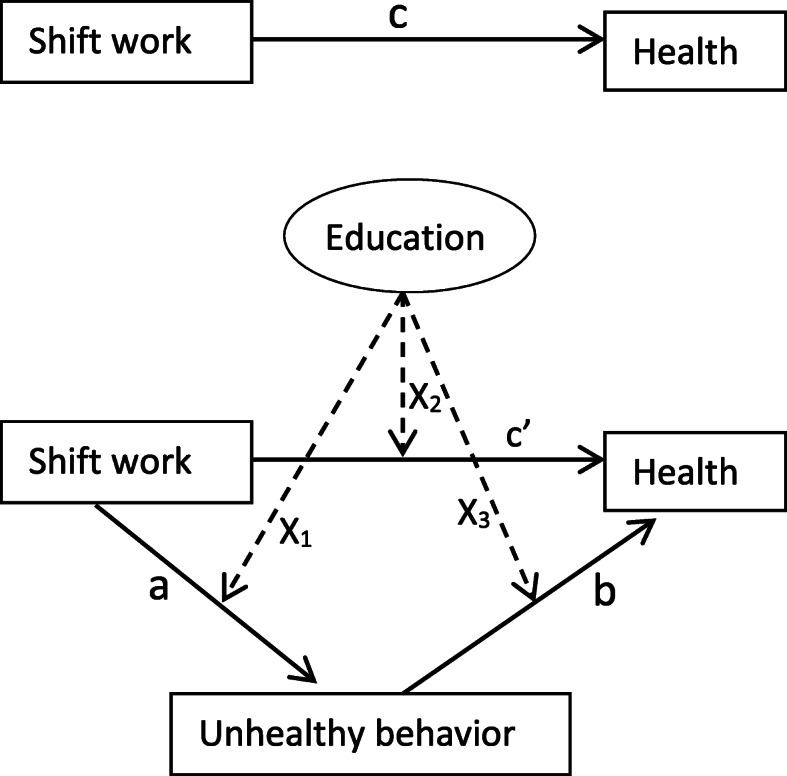
Schematic presentation of the total effects of shift work on mental or physical health (c), the indirect effects of unhealthy behavior (a, b), the direct effect of shift work on health (c’), and the moderation by education (x_1–3_), based on the moderated mediation framework of Edwards (2007)

Moderated mediation analyses were conducted based on the framework described by Edwards and Schurer Lambert (2007) [39]. Figure 1 shows a schematic presentation of the framework, applied to the relationship between shift work, unhealthy behavior, perceived health and education. The upper part shows the total effect (c) of shift work on perceived health. The lower part shows the mediation model of the indirect effect of unhealthy behavior (a, b), and the direct effect of shift work (c’) on perceived health that is independent from the mediators and other covariates. As we, used the health measurements from the third to sixth round, there was a 5-year time-lag between the measurements of unhealthy behavior and that of perceived health.

Following this framework, mediation was assessed by fitting two models. First, a model was fitted in which unhealthy behavior (potential mediator) was the dependent variable, and shift work was the independent variable. If a statistically significant association between shift work and unhealthy behavior was observed in this first model, a second model was tested. In this second model, both shift work and unhealthy behavior were included as independent variables and perceived health in the next round (5 years after the measurement for BMI or unhealthy behavior) was taken as the dependent variable. All models were adjusted for sex, age (continuous) and educational level.

In the first model, moderation was assessed by including an interaction term of shift work status and educational level. In the second model, moderation was assessed by including two interaction terms: shift work*educational level and unhealthy behavior*educational level. In case of a statistically significant interaction (*p*-value< 0.10), indirect effects of the mediation analysis were separately calculated for low and intermediate/high educational level.

The direct effect of shift work on perceived health is represented by the coefficient for shift work status in the second model. Indirect mediation effects were calculated using the product of coefficients method: the coefficient for shift work status in the first model was multiplied by the coefficient for unhealthy behavior in the second model. Indirect effects were tested by bootstrapping (1500 replications) of the entire procedure and constructing a confidence interval based on the 2.5th and 97.5th percentile. Total effects were calculated by taking the sum of the direct and indirect effects. Total effects were also tested using the bootstrap method. Generalized estimating equations (GEE) were used to estimate the coefficients. Because the product of coefficients method was used, linear models were used to estimate the coefficients in all models. SAS version 9.4 was used for all statistical analyses. Mediation was assessed first for all mediators separately, and subsequently a multivariate model was used combining relevant mediators.

## Results

Table 1 shows the characteristics of the study population, divided by shift work status. The mean age of the study population was 42.2 (sd 8.1) years, 40.7% were female and 40.0% had a low educational level. As to the unhealthy behaviors, 26.7% of the study population were smokers, 57.0% was physically inactive and 43.7% had overweight. Shift workers were on average younger (40.9 yr versus 42.4 yr), more often smokers (35.9% versus 25.5%) and less often physically inactive (50.3% versus 57.9%) than non-shift workers. Shift workers were mostly rotating shift workers (89.8%), some of them worked night (3.8%) or evening shifts (6.4%) only.

**Table 1:** Characteristics of the study population at baseline, separated for shift workers and non-shift workers

	All participants (*n* = 1633)	Shift workers (*n* = 195)	Non-shift workers (*n* = 1438)
Sex (% female)	40.7%	36.9%	41.2%
Age (mean, sd)	42.2 (8.1)	40.9 (8.4)	42.4 (8.0)
Education (% low)	40.0%	43.1%	39.5%
Years of shift work (mean, sd)	–	14.4 (8.9)	–
Mental health^a^ (mean, sd)	78.0 (14.1)	78.3 (12.1)	77.9 (14.3)
Physical functioning^a^ (mean, sd)	91.2 (13.0)	91.5 (11.6)	91.2 (13.2)
Smoking (% current smoker)	26.7%	35.9%	25.5%
Alcohol consumption (> 1 glass per day)	42.9%	42.0%	43.0%
Physical inactivity^b^	57.0%	50.3%	57.9%
BMI (mean, sd)	24.9 (3.1)	25.1 (3.1)	24.8 (3.1)
Overweight (≥25.0 kg/m^2^)	43.7%	45.6%	43.4%

^a^ Health scorings from 0 to 100, with a higher score indicating a better health

^b^ Physically active according to the guideline is defined as at least 30 min per day activities of moderate of high intensity

Shift workers were more likely to be overweight (prevalence of overweight 5% higher: B 0.05; 95% CI 0.01–0.10), or to smoke (prevalence of smoking 5% higher: B 0.05; 95% CI 0.01–0.09) and shift workers were less likely to be physically inactive (prevalence of being physically active 8% lower: B -0.08; 95% CI -0.14 – − 0.03) (Table 2). The association between shift work and alcohol consumption was weaker and not statistically significant, implying that alcohol consumption is not a mediator in the relationship between shift work and health, and therefore it was not tested as such.

**Table 2:** Associations (Beta’s and 95% confidence intervals) between shift work, BMI and unhealthy behavior

	Overweight	Smoking	Alcohol consumption	Physical inactivity
	B (95% CI) ^a^	B (95% CI) ^a^	B (95% CI) ^a^	B (95% CI) ^a^
Shift work (a)	**0.05 (0.01–0.10)**	**0.05 (0.01–0.09**)	0.03 (− 0.03–0.07)	**-0.08 (− 0.14 – − 0.03)**

^a^ Analyses were adjusted for sex, age and level of education, statistically significant results are highlighted in bold

### Mental health

Shift work and being overweight were not statistically significantly associated with mental health (Table 3). Smoking (B -1.89; 95% CI -2.98 – − 0.81) and physical inactivity (B -1.23; 95% CI -1.90 - -0.56) were significantly inversely associated with mental health. Overall, no statistically significant interaction between shift work or unhealthy behaviors and education was found (*p*-values x_1–3_ > 0.10), which indicated that estimates of indirect effects for low and intermediate/high educated workers did not differ enough to warrant separate analyses.

**Table 3:** Associations (Beta’s and 95% confidence intervals) between shift work or unhealthy behavior with mental health and physical functioning (*n* = 1633)

	Mental health^b^	Physical functioning^b^
	B (95% CI) ^a^	B (95% CI) ^a^
Shift work (c)	−0.11 (− 1.59–1.38)	− 0.88 (− 2.37–0.61)
Overweight (b)	0.64 (− 0.17–1.44)	**−0.97 (− 1.83 – − 0.12)**
Smoking (b)	**−1.89 (− 2.98 – − 0.81)**	**−1.55 (− 2.69 – − 0.42)**
Physical inactivity (b)	**−1.23 (− 1.90 – − 0.56)**	**−1.05 (− 1.81 – − 0.29)**

^a^ Analyses were adjusted for sex, age and level of education, statistically significant results are highlighted in bold

^b^ A higher score implies a better health

The mediation models showed that indirect effects on the relationship between shift work and mental health were present for smoking and physical inactivity (Table 4). Shift workers had a 0.09 (95% CI -0.20 – − 0.01) lower mental health score than non-shift workers via smoking (mediator), and a 0.11 (95% CI 0.03–0.23) higher mental health score via physical inactivity (mediator). The direct effect of shift work on mental health, controlling for the potential mediator (i.e. smoking or physical inactivity) was not statistically significant. Further analyses with all three potential mediators (overweight, smoking and physical inactivity) combined in one model, showed no statistically significant direct or indirect effect.

**Table 4:** Direct, indirect and total effects of shift work on mental health and physical functioning, with unhealthy behavior as potential mediator (*n* = 1633)

	Shift work – mental health	Shift work – physical functioning
	B (95% CI)	B (95% CI)
**Overweight**		
Total Effect^a^	−0.12 (− 1.59–1.30)	−0.86 (− 2.31–0.53)
Direct Effect^b^ (c’)	−0.15 (− 1.65–1.29)	−0.78 (− 2.22–0.76)
Indirect Effect^c^ (a*b)	0.03 (− 0.02–0.10)	− 0.08 (− 0.19–0.00)
**Smoking**		
Total Effect^a^	−0.11 (− 1.57–1.31)	−0.87 (− 2.25–0.50)
Direct Effect^b^ (c’)	−0.02 (− 1.50–1.45)	−0.78 (− 2.27–0.70)
Indirect Effect^c^ (a*b)	**−0.09 (− 0.20 – − 0.01)**	**−0.09 (− 0.21 – − 0.01)**
**Physical inactivity**		
Total Effect^a^	− 0.11 (−1.56–1.31)	−0.89 (− 2.30–0.49)
Direct Effect^b^ (c’)	−0.22 (− 1.70–1.26)	− 0.97 (− 2.46–0.52)
Indirect Effect^c^ (a*b)	**0.11 (0.03–0.23)**	**0.08 (0.01–0.18)**
**Overweight, smoking and physical inactivity**		
Total Effect^a^	−0.12 (− 1.59–1.30)	−0.88 (− 2.29–0.53)
Direct Effect^b^ (c’)	−0.17 (− 1.64–1.30)	−0.75 (− 2.25–0.74)
Indirect Effect^c^ (a*b)	0.05 (− 0.10–0.22)	− 0.13 (− 0.26–0.04)

^a^ Total effect of shift work on health, taking mediating effect into account

^b^ Direct effect of shift work on health, adjusted for the mediator, age, sex and level of education

^c^ Indirect effect of shift work on health through the mediator

### Physical health

Shift work was not statistically significantly associated with a lower physical health (Table 3). Overweight (B -0.97; 95% CI -1.83 – − 0.12), smoking (B -1.55; 95% CI -2.69 – − 0.42) and physical inactivity (B -1.05; 95% CI -1.81 - -0.29) were statistically significantly associated with a lower physical health. Educational level did not significantly moderate the relationship between shift work and physical health (*p*-values interaction terms x_1–3_ > 0.10).

Also for physical health, indirect effects for smoking and physical inactivity were found in the relationship between shift work and physical health (Table 4). Shift workers scored 0.09 (95% CI -0.21 – − 0.01) points lower via smoking (mediator), and 0.08 (95% CI 0.01–0.18) points higher than non-shift workers on physical health via physical inactivity (mediator). Direct effects of shift work on physical health were not statistically significant. When all three mediators (overweight, smoking and physical inactivity) were combined in one model, no statistically significant indirect effects were observed.

## Discussion

This study shows that shift work was not statistically significantly related with either mental or physical health. Shift workers are more likely to have overweight and to smoke compared to non-shift workers, which are both risk factors for poor physical functioning and smoking is also a risk factor for poor mental health. On the other hand, shift workers were more physically active than non-shift workers, which is related to better mental health and physical functioning. However, the mediation effects of the unhealthy behavior in the relationship between shift work and perceived health were very small.

We did not find a statistically significant relationship between shift work and mental health. Although some conclude that a lack of statistical significance in the relation between x and y excludes mediation, or at least renders it unlikely, this assumption is not always valid [40, 41]. In the view of Zhao and colleagues (2010), and that of others, a statistically significant direct effect or total effect is not a necessary condition for mediation to be present [41–43]. Indeed, statistically significant indirect effects were found and it can be concluded that the relationship between shift work and mental health is mediated by both smoking and physical inactivity. However, the indirect effect via physical activity was in the opposite direction to the direct effect and thus alleviated the negative direction of the direct effect of shift work on mental health. Interestingly, a recent study using the UK biobank also showed that shift workers had higher levels of physical activity, despite the fact that this shift work was associated cross-sectionally with depression [44].

As the direction of all the indirect effects of unhealthy behavior in the relationship between shift work and mental health were opposite to that of the direct effects, the direct and indirect effects canceled each other. In general, the magnitude of the indirect effects is very small: only a 0.09 point decrease in MHI-5 score through smoking and a 0.11 point increase through physical inactivity between shift workers and non-shift workers were observed. Considering the score range of 0–100, it can be concluded that there is no meaningful effect of shift work on mental health, neither directly nor through mediation.

Mediation of the relationship between shift work and physical health was present through smoking and physical inactivity, as indicated by the indirect effects. Similar to the findings for mental health, small differences between shift workers and non-shift workers were apparent with the largest effect being a 0.09 point decrease in the expected direction. Thus, despite the statistically significant mediating effect found, this effect does not seem to seem relevant for public health.

Although the potential mediating role of lifestyle behaviors has been hypothesized [21, 45], we could not retrieve any study into such a mediating role with regard to perceived health as outcome. A recent study by Hulsegge et al. revealed that shift workers had greater odds for obesity and diabetes which was mediated by poorer sleep quality. In addition, lower physical activity levels and lower intake of fruit and vegetables were also mediators in the relationship between shift work and obesity, but not in the relationship between shift work and diabetes [46]. For the association between shift work and respiratory infections, a mediating role of poor sleep quality was confirmed, but not of low physical activity or unhealthy diet [47].

From the perspective that educational level is related to each of the variables in the model (i.e. shift work, health, BMI and unhealthy behavior), this study also aimed to explore the moderating role of educational level (see Fig. 1). Our results did, however, not confirm such a role, neither for mental health nor for physical health. It should be mentioned that the present study focused on educational level as a measure of socioeconomic status, while the latter is a composite of not only education, but also of income, occupation, and other factors.

A main strength of this study is its longitudinal data collection over an extended follow-up period. Data on lifestyle and health were collected every 5 years with high response rates. Shift work was assessed retrospectively, which makes our results susceptible to information bias. However, we believe the participants were able to reliably recall which years during their life course they performed shift work, since being a shift worker has a significant impact on someone’s life. The choice for our statistical framework to determine mediation effects was guided by the question on the moderating role of education in the relationship between shift work, unhealthy behavior and perceived health. Stratified analyses in low and high educated workers were not feasible due to limited statistical power, which may have implications for tests of mediation in each subgroup [48]. The moderated mediation framework was applied and this approach uses the product of coefficients method to calculate indirect effects [48]. The product of coefficients method has been criticized because of the assumption that no significant interaction exists between the independent variable and the mediator [49], in our study between shift work and unhealthy behavior in the relationship with perceived health. For this reason, we tested interaction effects of shift work and unhealthy behaviors, which turned out to be non-significant (*p* > 0.10).

The choices we made in categorizing lifestyle exposures, which were based on public health recommendations [35, 37], may have implications for our findings. With regard to alcohol consumption, a cut-off for safe alcohol consumption was used. This may have caused an underestimation of a potential association between shift work and more harmful alcohol consumption. Also the findings for overweight using a cut-off of ≥25 kg/m^2^ may underestimate the association between shift work and more serious overweight or obesity, since other studies reported a stronger association with obesity [50]. A sensitivity analysis on our data showed that shift work was not statistically significantly associated with obesity, therefore a mediation analysis for obesity would not have been warranted. A final point of discussion is that our sample of shift workers seemed to be rather healthy, or very similar to day workers. This finding can probably be attributed to the healthy worker effect, those shift workers who experience the fewest health problems in general and health problems in doing shift work in particular, are most likely to remain in the shift work population [51] and this may have to some degree influenced our results.

## Conclusions

No statistically significant effects were found of shift work on mental health and physical functioning. Mediation effects of overweight, smoking and physical inactivity were shown but these were very small. As the evidence for a link between shift work and poor health outcomes is increasing, future research should further investigate the role of unhealthy behaviors as mediator of the relationship between shift work and perceived health.

## Data Availability

Due to ethical restrictions related to participant consent, all relevant data are available upon request to the principal investigator of the Doetinchem Cohort Study: professor WMM Verschuren (email: monique.verschuren@rivm.nl). More information can be found at: https://www.rivm.nl/doetinchem-cohort-studie/onderzoekers/aanvraag-gegevens-dcs.

## Abbreviations

BMI: Body Mass Index
SF-36: 36-item Short Form Health Survey
MHI-5: 5-item Mental Health Inventory
GEE: Generalized Estimating Equations
